# A rationally enhanced red fluorescent protein expands the utility of FRET biosensors

**DOI:** 10.1038/s41467-020-15687-x

**Published:** 2020-04-15

**Authors:** Gary C. H. Mo, Clara Posner, Erik A. Rodriguez, Tengqian Sun, Jin Zhang

**Affiliations:** 1 Department of Pharmacology, University of California San Diego, La Jolla, CA 92093 USA; 2 Department of Bioengineering, University of California San Diego, La Jolla, CA 92093 USA; 3 Department of Chemistry, The George Washington University, Washington, DC 20052 USA; 4 Department of Chemistry and Biochemistry, University of California San Diego, La Jolla, CA 92093 USA; 5 Present Address: Department of Pharmacology, University of Illinois at Chicago, Chicago, IL 60612 USA

**Keywords:** Fluorescence imaging, Fluorescence resonance energy transfer, Cell signalling

## Abstract

Genetically encoded Förster Resonance Energy Transfer (FRET)-based biosensors are powerful tools to illuminate spatiotemporal regulation of cell signaling in living cells, but the utility of the red spectrum for biosensing was limited due to a lack of bright and stable red fluorescent proteins. Here, we rationally improve the photophysical characteristics of the coral-derived fluorescent protein TagRFP-T. We show that a new single-residue mutant, super-TagRFP (stagRFP) has nearly twice the molecular brightness of TagRFP-T and negligible photoactivation. stagRFP facilitates significant improvements on multiple green-red biosensors as a FRET acceptor and is an efficient FRET donor that supports red/far-red FRET biosensing. Capitalizing on the ability of stagRFP to couple with multiple FRET partners, we develop a novel multiplex method to examine the confluence of signaling activities from three kinases simultaneously in single living cells, providing evidence for a role of Src family kinases in regulating growth factor induced Akt and ERK activities.

## Abstract (web summary)

TagRFP is a bright red fluorescent protein, but undergoes photoconversion to a dark state, making it undesirable for conventional fluorescence microscopy. Here, the authors introduce stabilising mutations to create super-TagRFP (stagRFP) and demonstrate its application as both a FRET acceptor and FRET donor.

## Introduction

The coral proteins TagRFP^1^ and a single residue mutant TagRFP-T^2^ are red fluorescent proteins (RFPs) that are twice as bright as the extensively used RFP mCherry. Despite high biophysical brightness and well-characterized chromophore mechanics, TagRFP and TagRFP-T are under-utilized due to high propensity for dark-state conversion^3^. This process causes fluorescence “blinking”, which is undesirable in conventional fluorescence microscopy. We showed that through a phenomenon called Fluorescence fLuctuation Increase by Contact (FLINC), the fluorescent protein Dronpa can specifically and quantitatively enhance the rate and probability of dark-state conversion of TagRFP-T. By controlling these fluctuations using conformational switches, we built a new class of fluorescent biosensors capable of biochemical activity imaging at super-resolution^4^ in live cells. This discovery has a noteworthy corollary: in the absence of dark-state processes such as FLINC, TagRFP-T could become brighter, more stable, and better suited for conventional microscopy modes, such as FRET biosensing. Our previous work revealed that FLINC is mediated by external residues on TagRFP-T. Therefore, we chose not to target the internal residues in contact with the chromophore, like prior fluorescent protein optimizations. In this communication, we demonstrate how rational re-engineering of solvent-exposed residues leads to a highly useful RFP with improved stability and brightness that enhances FRET sensing and multiplexed imaging. We thus showcase green-red FRET biosensors with a dynamic range comparable to that of cyan-yellow FRET biosensors and reveal the complex interaction between the kinases Src, Akt and ERK within the same single cell.

## Results

### TagRFP-T is improved by mutagenesis at an external residue

**Fig. 1:**
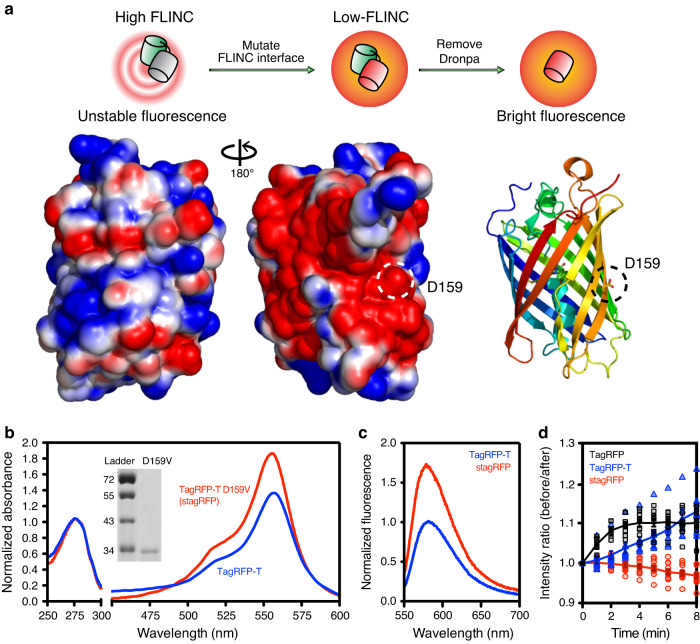
The rational mutagenesis model and the resulting enhanced spectroscopic characteristics of stagRFP compared to TagRFP-T. **a** Schematic underlying the rational engineering approach. A model system bearing high FLINC state reveals the presence of the external acidic residue that weakens the TagRFP-T chromophore. Site-directed mutagenesis products that rescue the model back to a low FLINC state harbor mutants with improved insulation and stable fluorescence. Finally, removal of Dronpa confirms the improved behavior of the mutant TagRFP-T. A ribbon structure and APBS electrostatic surface of TagRFP-T (based on PDB 3T6H) demonstrates the large acidic (red) solvent exposed surface opposite the chromophore. The location of D159 is indicated by dashed circles. Normalized in vitro absorption (**b**) and fluorescence (**c**) spectra of purified stagRFP compared to TagRFP-T demonstrating the significantly higher extinction coefficient and quantum yield. **d** Cyclic excitation of HeLa cells expressing different TagRFP variants show photoactivation is present in parent proteins (TagRFP and TagRFP-T), but not the new mutant stagRFP (TagRFP, *n* = 9 biologically independent cells; TagRFP-T, *n* = 10; and stagRFP, *n* = 12 biologically independent cells); each cell is represented by the, respectively, colored symbol, and the average values across all cells are shown using solid lines. Source data for **b**–**c** are provided as a Source Data file.

Mutagenesis confirmed positively-charged residues on Dronpa mediate FLINC by electrostatic interactions, using a fusion model where Dronpa is directly fused to TagRFP-T^4^. We hypothesized that removing acidic residues on the exterior of TagRFP-T would reduce FLINC and dark-state conversion to produce a more desirable RFP. The Dronpa-TagRFP-T fusion model provides a quantitative means for mutagenic screening, in which an improved TagRFP-T mutant will decrease dark-state conversion and increase red fluorescence intensity compared to the wild-type TagRFP-T (Fig. 1a). We performed random mutagenesis at 4 solvent-exposed, charged residues on TagRFP-T fused to Dronpa. Two acidic residues D159 and D196 were expected to decrease dark-state conversion, while two basic residues (R157 and R198) were chosen as the negative controls and should not change the blinking or increase fluorescence intensity. Libraries of single and multiple site mutations were generated using fully degenerate primers, protein was expressed in *E. coli*, and the RFP fluorescence intensity was quantified. After 4 rounds of screening, fluorescence intensity increase was not observed at residues R157, R198, or D196, suggesting these external positions do not contribute significantly to TagRFP-T fluorescence and/or dark-state conversion. However, several mutations at D159 yielded significant improvements. Consistent with our hypothesis, basic and bulky hydrophobic mutations (His, Phe, and Val) at D159 increased red fluorescence intensity, creating fusion proteins that are brighter than TagRFP-T alone. One mutant, D159V, was the most prevalent (>50% probability in 2 rounds of random mutagenesis). D159V retained bright fluorescence when expressed alone and not fused to Dronpa. It was named super-TagRFP (stagRFP) and further characterized.

Organized Smooth Endoplasmic Reticulum (OSER) assays indicated that stagRFP has a tendency to dimerize or oligomerize (Supplementary Fig. 1), similar to parent proteins TagRFP and TagRFP-T^5,6^. stagRFP contains the same number of tryptophan, tyrosine, and cysteine residues and same β-barrel fold as TagRFP-T. We thus examined freshly purified TagRFP-T and stagRFP and normalized the absorbance at 280 nm to directly compare their absorption spectra. While stagRFP absorption and fluorescence spectra remain similar to that of TagRFP-T, the extinction coefficient and fluorescence quantum yield of stagRFP were larger (Fig. 1b, c). Published TagRFP-T extinction coefficients^2^ allowed us to determine stagRFP extinction coefficient as 113,000 M^−1^ cm^−1^ and a quantum yield of 0.53, resulting in an apparent brightness index (extinction coefficient × quantum yield) of 59,000. Using plasma membrane targeted TagRFP, TagRFP-T, and stagRFP expressed in HeLa cells, we further examined the stability of stagRFP. TagRFP-T and TagRFP are known to display significant and irreversible photoactivation, especially using wide-band and blue-shifted excitation wavelengths used for GFP^3^. By using ratios of the stable red fluorescence intensity within each cycle of alternate excitation (I_after-GFP_/I_before-GFP_), we quantified the irreversible photoactivation of these FPs (Fig. 1d). Both parent proteins showed significant photoactivation. TagRFP is significantly and fully activated after a few cycles, while the apparent intensity of TagRFP-T continues to increase even after 10 cycles. stagRFP showed negligible photoactivation.

### stagRFP enhances FRET biosensing in the red spectrum

The green-red regions of the visible spectrum are favored for fluorescence imaging due to widely available excitation sources and relatively clean spectral separation. However, green-red genetically encoded FRET biosensors tend to have low dynamic range and are further complicated by the red chromophore maturation from green to red. The enhanced properties of stagRFP prompted us to examine whether it would facilitate a significant gain in dynamic range. We compared the performance of stagRFP as a FRET acceptor with commonly used RFPs in the context of GFP-RFP based A Kinase Activity Reporters (AKARs)^7^. First, we show the control biosensor that incorporated wild-type TagRFP-T displayed a complex ratiometric response, where the acceptor-direct fluorescence intensity steadily increased by over 50% due to TagRFP-T photoactivation and biased the ratiometric readout (Supplementary Fig. 2A). Substitution of TagRFP-T with stagRFP corrected this photoactivation artifact, making ratiometric FRET imaging straightforward (Supplementary Fig. 2B). The direct RFP fluorescence intensity in the stagRFP-based biosensor was stable and typically within 5% of initial intensity after more than 30 min of wide-field epifluorescence FRET imaging.

**Fig. 2:**
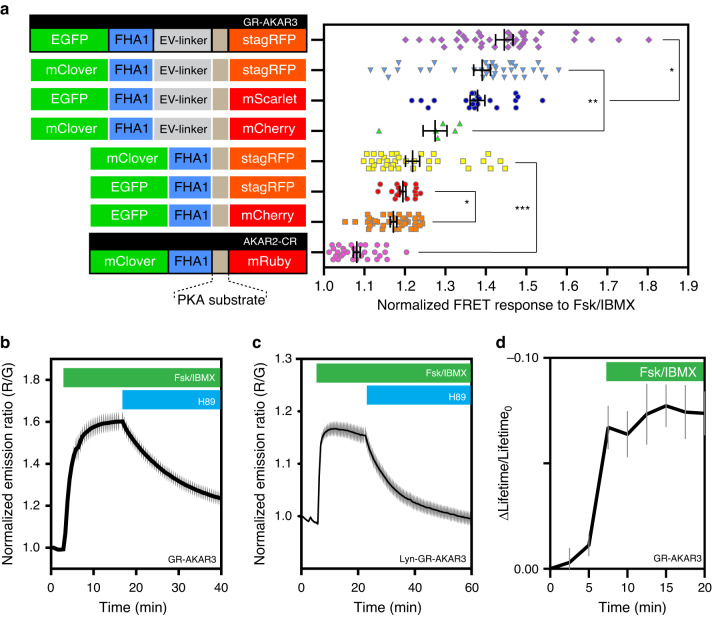
Comparison highlighting the improved dynamic range of green-red A Kinase Activity Reporters (AKARs) when utilizing stagRFP. **a** stagRFP confers a significant increase in FRET dynamic range. The mean ± SEM is displayed. Stimulation of PKA in HeLa or HEK293T cells was performed using 50 μM forskolin and 100 μM IBMX, while inhibition was performed using 20 μM H89. *p < 0.05; **p < 0.01; ***p < 0.0001 from two tailed, unpaired Welch’s *t*-test. Specifically, *p* values for the comparison between stagRFP and mScarlet in the AKARev context *p* = 0.0225 (*t* = 2.349, df = 55); between stagRFP and mCherry in the mClover-AKARev context *p* = 0.0089 (*t* = 3.239, df = 10); between stagRFP and mCherry in the EGFP-AKAR context *p* = 0.0430 (*t* = 2.080, df = 47); and between stagRFP and mRuby in the AKAR context *p* < 0.0001 (*t* = 15.80, df = 47). The changes in normalized emission ratio (red/green) are AKAR2-CR, 8.1 ± 0.8%, *n* = 32 biologically independent cells; EGFP-mCherry-AKAR, 17.2 ± 0.8%, *n* = 38; EGFP-stagRFP-AKAR, 19.5 ± 0.7%, *n* = 17; mClover-stagRFP-AKAR, 21.9 ± 1.7%, *n* = 35; mClover-mCherry-AKARev, 27.5 ± 2.9%, *n* = 6; EGFP-mScarlet-AKARev, 38 ± 1.8%, *n* = 21; mClover-stagRFP-AKARev, 39.1 ± 2.0%, *n* = 35; EGFP-stagRFP-AKARev, 44.6 ± 2.1%, *n* = 37. **b** Diffusible GR-AKAR3 displays a high dynamic range (*n* = 8). The mean ratio ± SEM is displayed. Source data are provided as a Source Data file. **c** Membrane-targeted biosensor is able to detect the increase in membrane PKA activity (*n* = 20). The mean ratio ± SEM is displayed. **d** The change in mean donor lifetime normalized to initial donor lifetime of GR-AKAR3 in HeLa cells upon Fsk/IBMX stimulation (*n* = 6). The mean ratio ± SEM is displayed in all panels.

In the development of cyan-yellow FRET biosensors, such as AKAR, further improvements were made by introducing a brighter FRET donor^8^ or a semi-flexible linker^9^. We therefore utilized different GFPs and examined whether the incorporation of a more efficient donor and an additional linker could further improve the response. Comparing the responses of several biosensor variants upon stimulation of PKA using forskolin (Fsk) and 3-isobutyl-1-methylxanthine (IBMX), stagRFP enabled a significant increase in dynamic range. The average response of PKA biosensors using stagRFP was significantly larger than the same biosensor with mCherry. This is true for the AKAR biosensor: (1) with an EGFP donor and short-linker, where stagRFP enabled a 19.5 ± 0.8% (mean ± SEM; *n* = 17 biologically independent cells) average response compared to 17.2 ± 0.8% (*n* = 38) using mCherry (*p* = 0.04, *t* = 2.080, df = 47 from two tailed, unpaired Welch’s *t*-test); or (2) using a mClover donor and semi-flexible linker, where stagRFP achieved an average response of 39.1 ± 2.1% (*n* = 35) compared to 27.5 ± 2.9% (*n* = 6) using mCherry (*p* = 0.009, *t* = 3.239 df = 10) (Fig. 2a). The best performing biosensor was named GR-AKAR3, with EGFP and stagRFP as the FRET pair and a semi-flexible linker (AKARev), displayed a further improvement resulting in an average response of 44.6 ± 2.1% (*n* = 37, maximum dynamic range of 80%) that is 6-fold better than the average response of 8.1 ± 0.9% (*n* = 32) of the current best green-red biosensor AKAR2-CR,^10^ utilizing the FRET pair mClover and mRuby, in the same cell lines (Fig. 2a). The new green-red GR-AKAR3 is comparable to the cyan-yellow PKA activity reporter AKAR4^8^.

We further compared the performance of stagRFP in green-red FRET-biosensing with the recently developed ultra-bright red fluorescent protein mScarlet^11^ in this optimized AKARev format, replacing the stagRFP acceptor in GR-AKAR3 with mScarlet. Both stagRFP and mScarlet enabled robust detection of PKA activity, with GR-AKAR3 being slightly better-performing (*p* = 0.02, *t* = 2.349, df = 55) than the mScarlet-based sensor (38.0 ± 1.8%, *n* = 21). GR-AKAR3 is reversible like the predecessor AKAR4 (Fig. 2b). When targeted to the plasma membrane, the average response of Lyn-GR-AKAR3 is 16.7 ± 1.5% (Fig. 2c, *n* = 20, maximum response of 32%) and comparable to that of the membrane-targeted, cyan-yellow PKA activity reporter Lyn-AKAR4^8^. In addition, the response of GR-AKAR3 can also be detected as a decrease in the mean donor (GFP) lifetime using Fluorescent Lifetime Imaging Microscopy (FLIM) (Fig. 2d) and as a decrease in the apparent mean acceptor fluorescence anisotropy using anisotropy imaging (Supplementary Fig. 3). Thus, stagRFP should facilitate the development of green-red FRET biosensors whose performance is on par with their cyan-yellow counterparts.

**Fig. 3:**
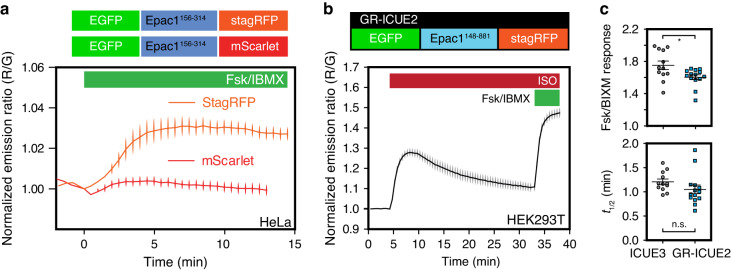
The cAMP detection capability of FRET biosensors using stagRFP is comparable to cyan-yellow biosensors. **a** Epac1-camps biosensor is utilized to demonstrate the advantage of stagRFP, where the same sensor design yields a viable FRET biosensor using stagRFP, but not using the ultra-bright RFP mScarlet. The change in normalized emission ratio (red/green) upon 50 μM forskolin and 100 μM IBMX stimulation is shown (stagRFP, *n* = 8 biologically independent cells; mScarlet, *n* = 11). The mean ratio ± SEM is displayed. **b** The response to adrenergic stimulation (1 μM isoproterenol) in HEK293T cells is captured by GR-ICUE2 at high dynamic range. The change in normalized emission ratio (green/red) is shown (*n* = 20). The mean ratio ± SEM is displayed. **c** The dynamic range of GR-ICUE2 is comparable to, but still significantly smaller (*p = 0.03, *t* = 2.399, df = 17, from two tailed, unpaired Welch’s *t*-test) than the best cyan-yellow cAMP biosensor ICUE3; upon Fsk and IBMX stimulation, the kinetics of GR-ICUE2 are not significantly different from those of ICUE3 (ICUE3, *n* = 12; GR-ICUE2, *n* = 14). The mean ratio ± SEM is displayed, and n. s. is not significant. Source data for **c** is provided as a Source Data file.

We next examined the performance of stagRFP in second messenger cAMP biosensors, starting with the molecular switch within Epac-camps^12^, which is known to have a limited conformational change due to the use of a single cAMP binding domain of Epac1 instead of the full-length protein^13^. We made Epac1-camps biosensors that utilized mScarlet or stagRFP as acceptors at the C-terminal. In this context, the mScarlet-based biosensor showed minimal response (0.3 ± 0.2%, *n* = 11), whereas the stagRFP counterpart showed a clearly significant FRET response (3.3 ± 0.4%, *n* = 8, *p* = 0.005, *t* = 3.642, df = 9) (Fig. 3a). Despite the limited dynamic range, this data demonstrates that the use of stagRFP in biosensors could help identify functional molecular switches. We next incorporated stagRFP into a biosensor that utilizes truncated Epac1 as the cAMP-dependent molecular switch to create a green-red variant of the cAMP biosensor ICUE^14^. Stimulation of HEK293T cells expressing the new GR-ICUE2 with isoproterenol (ISO), an adrenergic receptor agonist, showed that GR-ICUE2 could reversibly detect transient cAMP response (Fig. 3b). GR-ICUE2 displays an average FRET response of 60.5 ± 3.0% (*n* = 14; maximum dynamic range of 71.0%) in HeLa cells upon Fsk and IBMX stimulation, which is close to the best cyan-yellow cAMP biosensor ICUE3 (average response 75.1 ± 5.3%, *n* = 12; Fig. 3c top panel). The kinetics of cAMP increase upon Fsk and IBMX stimulation was also comparable to the currently most optimized version ICUE3^15^ (Fig. 3c, lower panel).

**Fig. 4:**
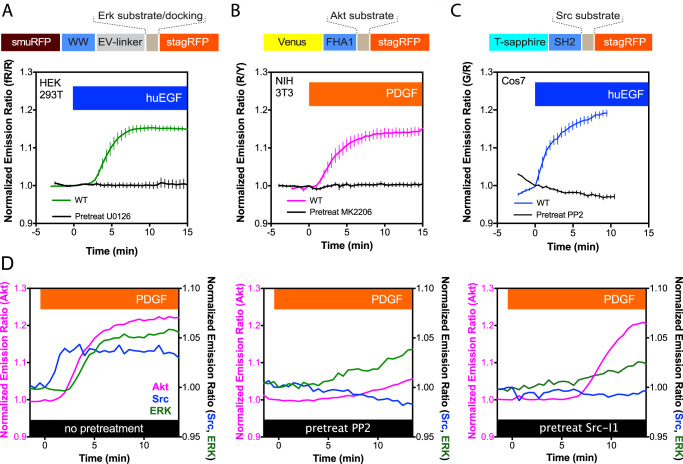
Multiplexing the kinase activities of Akt, Src, and ERK in single live cells utilizing three biosensors containing stagRFP. **a** The red-far-red biosensor fRR-EKARev could reliably monitor ERK activity in response to 100 ng/mL huEGF stimulation in the red-far-red region, shown as changes in normalized emission ratio (far-red/red) (WT, *n* = 12 biologically independent cells; and pretreatment with 20 µM U0126, *n* = 5). **b** The yellow-red Akt activity reporter incorporating stagRFP reliably detects Akt activity in NIH 3T3 cells in response to 50 ng/mL PDGF stimulation, shown as changes in normalized emission ratio (red/yellow) (WT, *n* = 6; and pretreatment with 3 µM MK2206, *n* = 8). **c** The green-red Src activity reporter incorporating stagRFP reliably detects Src activity in Cos7 cells in response to 100 ng/mL huEGF stimulation, shown as changes in normalized ratio (red/green) (green for T-sapphire emission) (WT, *n* = 6; and pretreatment with 10 µM PP2, *n* = 12). The mean ratio ± SEM is displayed for **a**–**c**. **d** Multiplexed imaging revealed a potential kinetic relationship between Src, Akt, and ERK upon 50 ng/mL PDGF stimulation. Representative curves showing PDGF-induced responses of green-red based Src activity biosensor (blue), yellow-red based Akt activity biosensor (red), and ERK activity biosensor (green) in control cells without pretreatment (left), cells pretreated with 10 µM PP2 (middle), or 10 µM Src-I1 (right).

The far-red and near-infrared portion of the visible spectrum was off-limits to FRET biosensing due to the lack of effective candidates for both an RFP donor and far-red acceptor. Recently, efforts have been made in creating FRET biosensors using two near-infrared FPs^16^. We note that FRET between an RFP and a far-red FP could prove advantageous for simultaneously imaging multiple sensors. A new bright, far-red FP incorporating the exogenous chromophore biliverdin and named the small Ultra-Red Fluorescent Protein (smURFP)^17^ should be useful as a FRET acceptor due to its large extinction coefficient of 180,000 M^−1^ cm^−1^. We demonstrate that stagRFP is an effective donor to the smURFP acceptor on an ERK biosensor scaffold^18^. In HEK293T cells, the new fRR-EKARev biosensor displays an average response of 15.1 ± 0.6% (*n* = 12, Fig. 4a) after human EGF (huEGF) stimulation (100 ng/mL); HEK293T cells pretreated with the MEK inhibitor U0126 (20 μM) showed little huEGF stimulated ERK response. The response of the new fRR-EKARev biosensor can also be detected as a decrease in the donor (stagRFP) fluorescence lifetime using FLIM (Supplementary Fig. 4) or as an apparent decrease in the acceptor fluorescence anisotropy using anisotropy imaging (Supplementary Fig. 5). These data demonstrate that stagRFP and smURFP form an efficient FRET pair. In brief, our data across different biosensing scenarios suggest that stagRFP is a highly effective FRET acceptor or donor and a useful alternative to current, bright RFPs.

### stagRFP supports multiplexed signaling dissection

To comprehensively decipher complex signal transduction pathways in living cells, we need to monitor not just the leading actor, but also a supporting cast of enzymes both upstream and downstream of a signaling node. Expanding the spectral range of FRET-based biosensors allows us to directly increase the number of biochemical activities one can multiplex and simultaneously observe. Biochemical experiments have shown that platelet-derived growth factor (PDGF) stimulation initiates a complex signaling cascade in NIH 3T3 fibroblasts involving Src, Akt, and ERK, which are nodal points of distinct survival/growth pathways. While these kinase activities have been monitored individually^18–20^, simultaneously imaging of all three has not been possible to observe the relative temporal hierarchy of triple kinase cascades in real time within the same cell. To address this question, we set out to test the multiplexed imaging of 3 different biochemical activities using fRR-EKARev with other stagRFP-bearing FRET biosensors within the same living cells.

We constructed new variants of the Akt activity reporter (AktAR) and Src activity reporter (SrcAR) biosensors utilizing spectrally orthogonal donors Venus and T-sapphire, respectively, with stagRFP as the shared acceptor (Fig. 4b, c) for co-imaging^21^. Both of these new biosensors performed as expected based on previous use of these molecular switches in single-construct FRET imaging (Fig. 4b, c and Supplementary Fig. 6). The above mentioned fRR-EKARev was utilized to monitor ERK. Using a sequential acquisition scheme, these probes allowed us to follow the complex kinase kinetic activation relationships in single living cells. In single NIH 3T3 cells, PDGF stimulation elicited a rapid response from Src, followed by Akt, and then ERK (Fig. 4d). These multiplexed observations were consistent with their equivalent from single biosensor experiments (Supplementary Fig. 6). We estimated that the onset of Src activity occurred on average 1.0 ± 0.6 min (*n* = 20) after PDGF stimulation, whereas the beginning of Akt and ERK kinase activity was registered to 3.4 ± 2.1 min (*n* = 21) and 3.4 ± 2.2 min (*n* = 11) after stimulation, respectively (Supplementary Fig. 7A; individual curves in Supplementary Fig. 8). The kinetics of Src activity rise is significantly faster on average (*t*_1/2_ = 4.0 ± 2.3 min, *n* = 20; *p* = 0.005, *t* = 2.948, df = 38.35) than those of Akt (*t*_1/2_ = 6.3 ± 2.8 min, *n* = 21). The kinetics of ERK activity rise were more variable in these cells (*t*_1/2_ = 6.3 ± 3.0 min, *n* = 11), and many cells did not show a strong ERK response (Supplementary Fig. 8). Using this multiplex platform, we showed that this fast-cascade system is hampered by selective Src family kinase inhibitors, PP2 and Src-l1, prior to PDGF stimulation (Fig. 4d; individual curves in Supplementary Figs. 9 and 10). Under these conditions, Akt and ERK activities were markedly slower and, in some cases, largely dampened in amplitude. This hints at a built-in hierarchy to PDGFR signaling in NIH 3T3 cells. While making these observations using separate biosensors in different cells, cell-cell variations in receptor and kinase expression would introduce population-level error and mask the kinetic and temporal relationship between these kinases. Our multiplexing approach removes such ambiguity and directly examines the inter-kinase activity relationship as they occur in each single cell, demonstrating a more holistic platform for deciphering complex signaling cascades.

## Discussion

In this work, we utilized a single rational mutation to enhance TagRFP-T, leading to the development of stagRFP. The successful D159V mutation is directly C-terminal to the S158T mutation in TagRFP-T^2^, but on the external face of the β-barrel. We surmise that in TagRFP and TagRFP-T, D159 acts as a charged “lever” to the internal environment, permitting the β-barrel and chromophore in turn to be perturbed externally by solvent and protein-protein interactions. D159V is a novel example of an external residue that directly contributes to chromophore function. Beyond charge neutralization, the valine modification may provide hydrophobic spatial bulk and limit hydrophilic solvent accessibility to the chromophore. The engineering considerations and insights, reported here, should inform the study of similar residues on other β-barrel fluorescent proteins, to potentially reveal previously overlooked properties.

We also developed a multiplexed imaging approach for analyzing three signaling activities in single living cells. Quantitative temporal relationship between different signaling events provides critical insights into signal propagation along a signaling cascade or dynamic cross-regulation between different pathways. However, this information is difficult to obtain from population-based biochemical assays owing to their limited temporal resolution. On the other hand, monitoring one signaling activity at a time in single-cell imaging and analyzing temporal correlation from parallel experiments can also present challenges because of cell-to-cell variations arising from differences in the expression of critical signaling components, such as membrane receptors. Multiplexed imaging simultaneously captures the precise temporal relationship between multiple signaling activities in a single living cell and provides a valuable tool to investigate the complex interplays.

Our visualization of the triple kinase activities following PDGFR stimulation in living NIH 3T3 cells illustrates the complexity of receptor signaling and suggests a cascade potentiated by Src family kinases, where several signaling branches, such as Akt/mTORC1 and Ras/MEK/ERK, may be modulated by these nonreceptor tyrosine kinases. The relationships between Src and these branches have been reported but not directly interrogated simultaneously in the same cells. For example, Src was shown to mediate hormone (such as estrogen) signaling by directly activating PI3K/Akt through phosphorylation of the PI3K regulatory subunit p85^22,23^. Src family kinases can also phosphorylate Grb2-associated-binders (Gab) family members, providing binding sites for multiple effector proteins, such as Src homology-2 (SH2)-containing protein tyrosine phosphatase 2 (SHP2) and p85. Although the interaction between Ras/Raf and Src is multi-layered^24^, Src can directly phosphorylate Raf to activate downstream MAP kinases, such as ERK^25,26^. Using a relatively selective inhibitor for Src family kinases PP2^27,28^ and an alternative dual competitive inhibitor for ATP and the substrate binding sites Src Inhibitor-1 (Src-I1)^29,30^, we show that Src family kinases significantly modulate the landscape of downstream kinases in PDGFR signaling. Future work will further dissect the underlying mechanisms and analyze the temporal activation to examine whether, similar to EGF/NGF signaling in PC12 cells^31^, NIH 3T3 fibroblasts can also actively encode distinct receptor signaling through kinetic kinase activation.

The multiplexed imaging method, demonstrated here, is complementary to other approaches, such as homoFRET anisotropy imaging and single-color (intensity-based) biosensing, each with associated benefits and disadvantages. HomoFRET detection requires specialized polarization optics and analysis and has limited dynamic range, but provides very rapid kinetics^32^. Single-color intensity sensors are laborious to develop and frequently sensitive to unintended responses, such as pH, but require the simplest microscopy instrumentation and regularly provide the largest dynamic range. In comparison, our development of stagRFP enhances quantitative ratiometric FRET imaging, making stagRFP generally applicable to any combination of FRET biosensors without the extensive optimization required for single-color sensors. Our data suggest that stagRFP is a useful FRET donor and acceptor, making stagRFP a crucial addition to the genetically encoded FP FRET toolkit. In the absence of such an RFP, biosensing is limited to utilizing multiple RFP species where inseparable cross-talk contaminates all FRET readouts involved. The TagRFP chromophore has a large 2-photon absorption cross-section at a region where its one-photon extinction is low^33^, making it a useful marker in 2-photon fluorescence imaging. The spectral similarity between stagRFP and its parent FP suggests that this characteristic could have been retained. Future work will examine 2-photon properties of stagRFP both in vitro and in cells, followed by 2-photon imaging of fRR-EKARev in cells and tissues. We expect that the new red-far-red FRET pair we developed should facilitate studies of the spatiotemporal activation of ERK and other kinases in vivo. In conclusion, we have demonstrated the rational improvement of TagRFP-T to create the bright stagRFP and showcased the improvements stagRFP contributes to FRET biosensors and multiplex imaging. The prevalence of many existing FRET molecular switches permits new sets of biochemical activities to be multiplexed simply by incorporating the FP pairings we reported here.

## Methods

### Mutagenesis

All site-directed mutagenesis experiments were performed following the protocol of Sawano and Miyawaki^34^ using the *E. coli* DH5α strain and plated onto LB-Amp agar. Random mutagenic primers were fully degenerate at the R157, D159, D196, and R198 residues. The TagRFP-T mutants were screened by colony fluorescence and only brightly fluorescent colonies (in both GFP and RFP channels) were sequenced. The mean intensity of each candidate in the RFP color channel was measured using an in-house fluorescence imager, illuminated by a broad-spectrum lamp source (MAX-303, Asahi Spectra) and monitored by a Thorlabs USB digital camera mounted behind a Thorlabs emission filter wheel for different fluorescence colors (CFP: 430/40; GFP: 535/40; and RFP: 630/75 nm). The images were analyzed using ImageJ (v. 1.47 g or later).

### Protein purification and characterization

TagRFP-T and stagRFP were over-expressed in the JM109 strain using pRSET-B vector at 37 °C then subsequently purified via His-tag affinity chromatography on separate Ni-NTA columns (Qiagen). The proteins were eluted using an imidazole gradient in 20 mM increments up to 200 mM imidazole. Pure fractions were concentrated via Amicon Ultra-15 filters (10,000 NMWL), stored at 4 °C in 50 mM Tris buffer with 300 mM NaCl at pH 7.4, and characterized within one month. Semi-native PAGE of stagRFP was performed using 10% polyacrylamide gels on non-boiled protein samples with minimal SDS content and stained using Coomassie Blue. Purified fluorescent proteins were simultaneously characterized by absorption (Beckman DU-650 spectrophotometer) and fluorescence (Horiba Jobin Yvon FluoroMax-3 fluorimeter). The absorbance spectra were normalized to the respective absorbance at 280 nm and the fluorescence spectra were normalized to the fluorescence maximum of TagRFP-T.

### Biosensor cloning

Cloning and subcloning were performed using the DH5α strain of *E. coli*. All mammalian constructs were cloned into the pcDNA3.0 vector with a modified multiple cloning site and all bacterial constructs were cloned into the pRSET-B vector. Plasmids were generated as follows: For GR-AKAR3 and variants, biosensor inserts were first subcloned into the pRSET-B vector using the restriction enzyme (RE) sites BamHI and EcoRI; the C-terminal fluorescent protein was then replaced by a PCR amplified stagRFP or mScarlet using primers encoding a 5’ SacI site and a 3’ EcoRI site; the donor position was then replaced by a PCR amplified EGFP using primers encoding a 5’ BamHI site and a 3’ BglII site. The green-red biosensor was subcloned into the pcDNA3.0 vector. Subsequent new biosensors (AktAR, SrcAR, and EKAR) employing the desired green-red FRET pairing were generated by replacing the AKAR biosensors with the target green-red pair using a PCR amplified fragment of the sensing unit alone using primers encoding a 5’ BglII site and a 3’ SacI site while in the context of pRSET-B, and subsequently subcloned into the pcDNA3.0 vector. In cases where these RE sites were not unique, site directed mutagenesis was utilized to obtain a unique combination or other RE sites, such as KpnI, were encoded instead. fRR-EKARev was generated by following a very similar protocol but placing smURFP at the N-terminal fluorescent protein position. Primer sequences are in Supplementary Table 1.

### OSER assay

pCytERM-mScarlet was a gift from Dorus Gadella (85066, Addgene), and CytERM-stagRFP was created by replacing the mScarlet with a PCR amplified stagRFP using primers encoding a 5’ AgeI site and a 3’ NotI site. OSER assay imaging was performed on a Nikon A1 confocal microscope. Hela cells at ~70% confluency were transfected with 500 ng of CytERM-FP constructs using Lipofectamine 2000 and incubated for 24 h before imaging. ImageJ was used to measure mean fluorescence intensity (MFI) of each OSER structure. Each cell nuclear envelope (NE) MFI was averaged from three MFI measurements from different edges of the nuclei, excluding any karmellae regions. Cells containing lobed or indistinct nuclei were excluded.

### Cell culture

The HeLa, HEK293T, and NIH 3T3 cells were maintained in DMEM media supplemented with 10% FBS and 1% penicillin and streptomycin. All cells were transfected at ~70% confluency using Lipofectamine 2000 and incubated for 24 h before imaging. NIH 3T3 cells were serum-starved to optimize growth-factor stimulation. Pretreatment of cells, to validate kinase biosensors, was performed by incubating cells in HBSS buffer with inhibitors at 37 °C for 15 min prior to imaging at the following concentrations. For ERK: HEK293T cells were treated with 20 µM U0126; for Akt: NIH3T3 cells were treated with 3 µM MK2206; for Src: Cos7 cells were treated with 10 µM PP2; and NIH 3T3 cells with 10 µM PP2 or Src-I1. All cells were imaged in HBSS buffer at room temperature. Cells transfected with fRR-EKARev biosensor were incubated with 12.5 μM biliverdin for 30 min in HBSS buffer at 37 °C before imaging.

### Fluorescence microscopy

All epifluorescence imaging was performed on a Zeiss Axiovert 200 M, AxioObserver Z1 Microscope, or AxioObserver Z7 equipped with a xeon lamp, a Sutter Lambda 10-3 that controls two separate filter wheels, a cooled (or cooled electron-multiplying) CCD, and examined under a 40X oil immersion objective. FRET microscopy of GFP/RFP biosensors was performed using the following excitation (EX) and emission (EM) filter combinations (maxima/bandwidth in nm): GFP–EX 480/30, EM 535/45, D505; RFP–EX 568/55, EM 653/95, D600; and FRET: EX 480/30, EM 653/95, D505. Emission intensities of individual cells were background-subtracted, and the ratios between the donor and FRET channel were normalized to the timepoint just before addition of stimuli (*t* = 0 min).

### Multiplex imaging

Co-imaging of 3 FRET biosensors was performed using either Multiplexed Filter Set A or Multiplexed Filter Set B. Multiplexed Filter Set A contains the following filter combinations (maxima/bandwidth in nm, dichroic (D) cutoff in nm): T-sapphire-RFP FRET–EX 380/10, EM 653/95, and D505; YFP-RFP FRET–EX 495/10, EM 653/95, and D515; smURFP-RFP FRET–EX 555/25, EM 700/75, and D660; RFP–EX 555/25, EM 653/95, and D600; T-sapphire–EX 380/10, EM 535/45, and D505; and YFP–EX 495/10, EM 535/25, and D515. Multiplexed Filter Set B contains the following filter combinations (maxima/bandwidth in nm, dichroic (D) cutoff in nm): T-sapphire-RFP FRET–EX 380/10, EM 645/75, and D505; YFP-RFP FRET–EX 495/10, EM 645/75, and D515; smURFP-RFP FRET–EX 572/35, EM 700/75, and D594; RFP–EX 572/35, EM 653/95, and D594; T-sapphire–EX 380/10, EM 535/50, and D505; and YFP–EX 495/10, EM 535/25, and D515. All FRET data was acquired using MetaFluor software and subsequently analyzed using MetaFluor software or in-house ImageJ macro programs and MATLAB scripts.

### Photoactivation studies

To enable photoactivation studies, cyclic excitation imaging was performed under total-internal-reflection fluorescence (TIRF) conditions using HeLa cells expressing plasma membrane-targeted RFP on a Nikon N-STORM/TIRF microscope equipped with an Andor IXON3 Ultra DU897 EMCCD camera, a Melles Griot argon laser (green-red FRET excitation: 488 nm), and a Coherent Sapphire solid-state laser (RFP direct excitation: 561 nm). Green-red FRET and then RFP direct images were taken at 100 ms exposures every 45 s with gain of 100, 10% setting on 488 nm power, and 1% setting on 561 nm power.

### Fluorescent lifetime imaging microscopy

FLIM was performed at 100× magnification on a Leica SP8 FALCON confocal microscope equipped with a white light laser and a Lecia HyD SMD detector. The laser was tuned to 500–530 nm for excitation of GR-AKAR-3 and tuned to 570–610 nm for excitation of fRR-EKARev and pulsed at a frequency of 80 MHz. Time course images were acquired every 2.5 min. Intensity weighted mean fluorescence lifetimes were calculated in LAS X (Lecia) software by fitting a bi-exponential decay model to the decay of each ROI. One ROI was selected per cell. Hela cells transfected with fRR-EKARev were serum starved overnight and incubated with HBSS and 12.5 μM biliverdin at room temperature for 30 min before performing FLIM.

### Anisotropy microscopy

Fluorescence anisotropy imaging was performed using a ×20 0.45 NA objective lens on a Zeiss AxioObserver. A polarizer (Chroma) was placed in the excitation pathway between the xenon arclamp and the excitation filters. Emission polarizations were separated using an Opto-Split II LS image splitter, with two wire grid polarizers (Meadowlark) oriented perpendicular and parallel to the excitation polarizer. The Hamamatsu Flash 4.0 sCMOS camera collected images containing both polarizations. FRET microscopy of GR-AKAR-3 and fRR-EKARev were performed using the following excitation (EX) and emission (EM) filter combinations (maxima/bandwidth in nm): GR-AKAR-3 FRET: EX 480/30, EM 653/95, D505 and fRR-EKARev FRET: EX 568/55, EM 700/75, D600.

Image analysis was performed using Fiji (ImageJ). Polarization images were cropped, background subtracted using Fiji’s built-in Rolling Ball Background Subtraction function, and then aligned using Fiji’s built-in StackReg function. Regions of Interest (ROIs) were manually selected in Fiji and their fluorescence emission intensities in both polarizations were measured. The anisotropy of each ROI was calculated using the equation,

$$r = \frac{{I_\parallel - g \cdot I_ \bot }}{{I_\parallel + 2 \cdot g \cdot I_ \bot }}$$

where $I_\parallel$ and $I_ \bot$ are the background subtracted fluorescence intensities in the parallel and perpendicular polarizations, respectively, and g is the correction factor accounting for differences in polarization emission efficiencies within the instrument. The g-factor was calculated using an isotropic fluorescein solution^35^. The change in anisotropy for each timepoint was calculated by subtracting the anisotropy value at the timepoint right before drug stimulation from the anisotropy calculated for that timepoint. Hela cells were transfected at ~70% confluency using Lipofectamine 2000 one day before imaging. Hela cells transfected with fRR-EKARev were serum starved overnight and incubated with HBSS and 12.5 μM biliverdin at 37 °C for 30 min before performing anisotropy imaging.

### Statistical methods

The reported statistical significance between control and experimental datasets were the results of two tailed, unpaired Welch’s *t*-tests calculated at 95% confidence level using Graphpad Prism.

### Reporting summary

Further information on research design is available in the Nature Research Reporting Summary linked to this article.

### Supplementary information

Supplementary Information

Reporting Summary

### Source Data

Source Data

## Data Availability

The source data underlying visible spectra and bar graphs in Figs. 1–3 and Supplementary Figs. 1 and 7 are provided as a source data file. The plasmids utilized in this article will be submitted to the non-profit plasmid repository, Addgene, for scientific sharing. All other data supporting the findings of this study, including the MetaFluor pseudocode/protocol are available upon reasonable request.

## References

[1] Merzlyak EM (2007). Bright monomeric red fluorescent protein with an extended fluorescence lifetime. Nat. Methods.

[2] Shaner NC (2008). Improving the photostability of bright monomeric orange and red fluorescent proteins. Nat. Methods.

[3] Dean KM (2011). Analysis of red-fluorescent proteins provides insight into dark-state conversion and photodegradation. Biophys. J..

[4] Mo GCH (2017). Genetically encoded biosensors for visualizing live-cell biochemical activity at super-resolution. Nat. Methods.

[5] Costantini LM, Fossati M, Francolini M, Snapp EL (2012). Assessing the tendency of fluorescent proteins to oligomerize under physiologic conditions. Traffic.

[6] Cranfill PJ (2016). Quantitative assessment of fluorescent proteins. Nat. Methods.

[7] Zhang J, Ma Y, Taylor SS, Tsien RY (2001). Genetically encoded reporters of protein kinase A activity reveal impact of substrate tethering. Proc. Natl Acad. Sci. USA.

[8] Depry C, Allen MD, Zhang J (2011). Visualization of PKA activity in plasma membrane microdomains. Mol. Biosyst..

[9] Komatsu N (2011). Development of an optimized backbone of FRET biosensors for kinases and GTPases. Mol. Biol. Cell.

[10] Lam AJ (2012). Improving FRET dynamic range with bright green and red fluorescent proteins. Nat. Methods.

[11] Bindels DS (2017). mScarlet: a bright monomeric red fluorescent protein for cellular imaging. Nat. Methods.

[12] Nikolaev VO, Bünemann M, Hein L, Hannawacker A, Lohse MJ (2004). Novel single chain cAMP sensors for receptor-induced signal propagation. J. Biol. Chem..

[13] Klarenbeek J, Goedhart J, Van Batenburg A, Groenewald D, Jalink K (2015). Fourth-generation Epac-based FRET sensors for cAMP feature exceptional brightness, photostability and dynamic range: characterization of dedicated sensors for FLIM, for ratiometry and with high affinity. PLoS ONE.

[14] DiPilato LM, Cheng X, Zhang J (2004). Fluorescent indicators of cAMP and Epac activation reveal differential dynamics of cAMP signaling within discrete subcellular compartments. Proc. Natl Acad. Sci. USA.

[15] Dipilato LM, Zhang J (2009). The role of membrane microdomains in shaping beta2-adrenergic receptor-mediated cAMP dynamics. Mol. Biosyst..

[16] Shcherbakova DM, Cammer NC, Huisman TM, Verkhusha VV, Hodgson L (2018). Direct multiplex imaging and optogenetics of Rho GTPases enabled by near-infrared FRET. Nat. Chem. Biol..

[17] Rodriguez EA (2016). A far-red fluorescent protein evolved from a cyanobacterial phycobiliprotein. Nat. Methods.

[18] Tang S, Yasuda R (2017). Imaging ERK and PKA activation in single dendritic spines during structural plasticity. Neuron.

[19] Wang Y (2005). Visualizing the mechanical activation of Src. Nature.

[20] Zhou X (2015). Dynamic visualization of mTORC1 activity in living cells. Cell Rep..

[21] Aye-Han N-N, Allen MD, Ni Q, Zhang J (2012). Parallel tracking of cAMP and PKA signaling dynamics in living cells with FRET-based fluorescent biosensors. Mol. Biosyst..

[22] Cuevas BD (2001). Tyrosine phosphorylation of p85 relieves its inhibitory activity on phosphatidylinositol 3-kinase. J. Biol. Chem..

[23] Haynes MP (2003). Src kinase mediates phosphatidylinositol 3-kinase/Akt-dependent rapid endothelial nitric-oxide synthase activation by estrogen. J. Biol. Chem..

[24] Bunda S (2014). Src promotes GTPase activity of Ras via tyrosine 32 phosphorylation. Proc. Natl Acad. Sci. USA.

[25] Marais R, Light Y, Fpaterson H, Marshall CJ (1995). Ras recruits Raf-1 to the plasma membrane for activation by tyrosine phosphorylation. EMBO J..

[26] Ziogas A, Moelling K, Radziwill G (2005). CNK1 is a scaffold protein that regulates Src-mediated Raf-1 activation. J. Biol. Chem..

[27] Hanke JH (1996). Discovery of a novel, potent, and Src family-selective tyrosine kinase inhibitor: study of Lck- and FynT-dependent T cell activation. J. Biol. Chem..

[28] Karni R (2003). The pp60c-Src inhibitor PP1 is non-competitive against ATP. FEBS Lett..

[29] Tian G, Cory M, Smith AA, Knight WB (2001). Structural determinants for potent, selective dual site inhibition of human pp60 c-src by 4-anilinoquinazolines. Biochemistry.

[30] Bain J (2007). The selectivity of protein kinase inhibitors: a further update. Biochem. J..

[31] Ryu H (2015). Frequency modulation of ERK activation dynamics rewires cell fate. Mol. Syst. Biol..

[32] Ross, B. L. et al. Single-color, ratiometric biosensors for detecting signaling activities in live cells. *Elife* **7**, e35458 (2018). 10.7554/eLife.35458 PMC6037473 29968564

[33] Drobizhev M, Makarov NS, Tillo SE, Hughes TE, Rebane A (2011). Two-photon absorption properties of fluorescent proteins. Nat. Methods.

[34] Sawano A, Miyawaki A (2000). Directed evolution of green fluorescent protein by a new versatile PCR strategy for site-directed and semi-random mutagenesis. Nucleic Acids Res..

[35] Piston DW, Rizzo MA (2008). FRET by fluorescence polarization microscopy. Methods Cell Biol..

