# Author Correction: A rationally enhanced red fluorescent protein expands the utility of FRET biosensors

**DOI:** 10.1038/s41467-024-45330-y

**Published:** 2024-01-26

**Authors:** Gary C. H. Mo, Clara Posner, Erik A. Rodriguez, Tengqian Sun, Jin Zhang

**Affiliations:** 1 Department of Pharmacology, University of California San Diego, La Jolla, CA 92093 USA; 2 Department of Bioengineering, University of California San Diego, La Jolla, CA 92093 USA; 3 Department of Chemistry, The George Washington University, Washington, DC, 20052 USA; 4 Department of Chemistry and Biochemistry, University of California San Diego, La Jolla, CA 92093 USA; 5 Present Address: Department of Pharmacology, University of Illinois at Chicago, Chicago, IL 60612 USA

Correction to: *Nature Communications* 10.1038/s41467-020-15687-x, published online 15 April 2020

The original version of this Article contained an error in Fig. 4c, in which y-axis label of “Normalized Emissions Ratio” was incorrectly given as “R/G”. The correct label should be “G/R”. This has been corrected in both the PDF and HTML versions of the Article.

