# Supplementary material for: A rationally enhanced red fluorescent protein expands the utility of FRET biosensors
Source: Nat Commun. 2020 Apr 15;11:1848. doi: 10.1038/s41467-020-15687-x (PMC7160135; doi:10.1038/s41467-020-15687-x)
Supplement: Supplementary file 1 — Supplementary Information [file 41467_2020_15687_MOESM1_ESM.pdf]

# A Rationally Enhanced Red Fluorescent Protein Expands the Utility of FRET Biosensors

**Mo et al.**

**Supplementary Information**

## Supplementary Figure 1

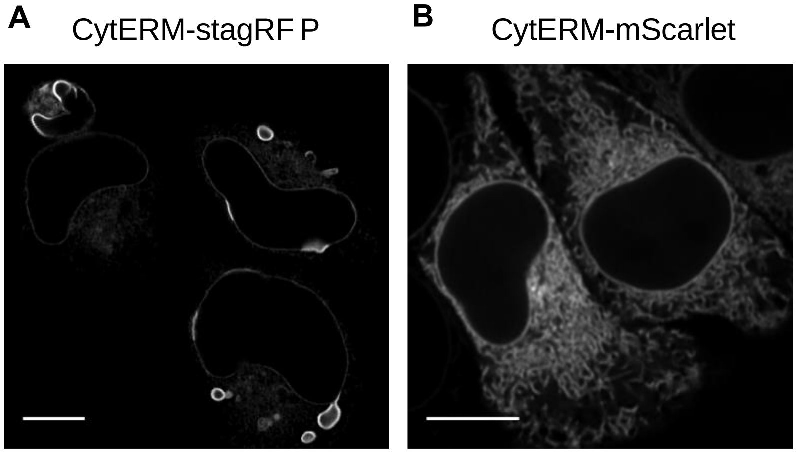


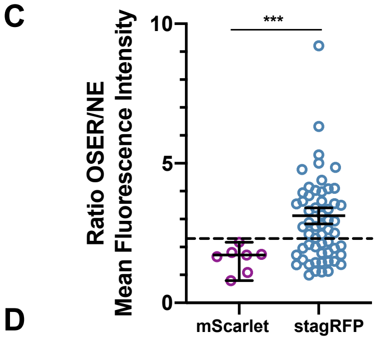


D

| | mScarlet | stagRFP |
|---|---|---|
| Total Cells | 29 | 23 |
| # OSER Structures | 7 | 59 |
| Percent Normal Cells | 82.8% | 30.4% |

**Supplementary Figure 1.** Assessment of oligomerization tendency of stagRFP compared to the monomeric FP, mScarlet, by the OSER Assay.[1,2] Representative images of CytERM-stagRFP (A) and CytERM-mScarlet (B) expressed in Hela cells. Scale bars are 10 µm. (C) Each dot represents a ratio of a whorl (OSER) structure's mean fluorescence intensity (MFI) over the nuclear envelope (NE). The mean ratio ± SEM is displayed (from stagRFP; n = 23 biologically independent cells; and mScarlet, n = 29). Dotted line at 2.3 represents the monomeric threshold. *** $p < 0.0001$, $t = 4.602$, $df = 46.17$; two tailed, unpaired Welch's t-test. (D) Table of OSER assay results. Cells displaying reticular ER and no OSER structures or lobed nuclei were considered normal. Source data are provided as a Source Data file.

**Supplementary Figure 2**

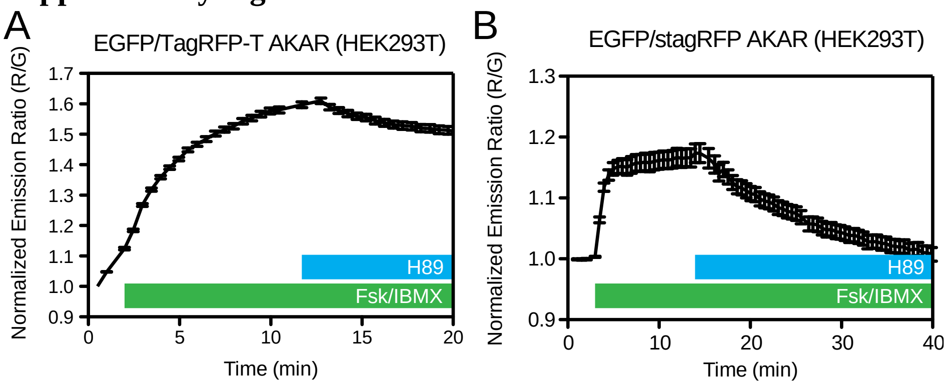


**Supplementary Figure 2**. Responses of PKA activity biosensors using photoactivatable TagRFP-T and photostable stagRFP acceptors upon sequential PKA stimulation and inhibition treatments. (A) EGFP/TagRFP-T AKAR displays clear photoactivation (n = 11 biologically independent cells). (B) EGFP/stagRFP AKAR (n = 16) shows no photoactivation. The photoactivation in (A) clearly prevents proper FRET sensor kinetics. The mean ratio ± SEM is displayed in both (A) and (B).

**Supplementary Figure 3**

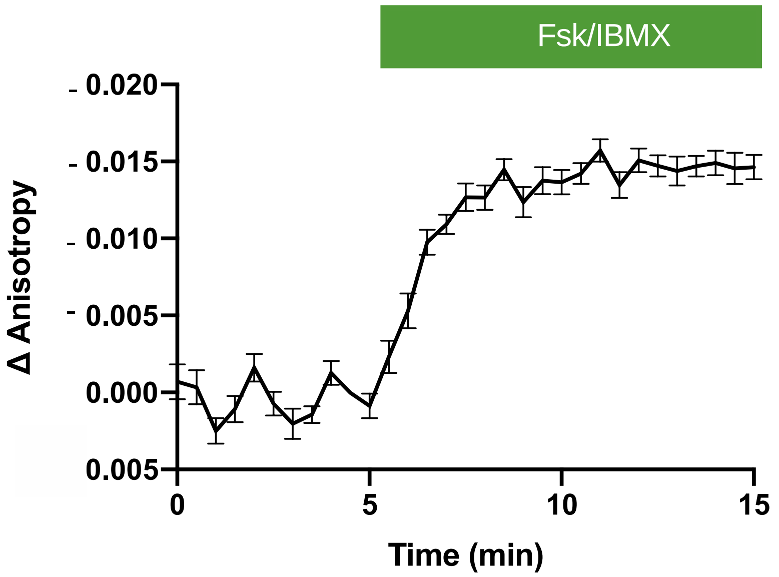


**Supplementary Figure 3.** Response of the GR-AKAR3 biosensor is detected as an apparent decrease in the acceptor anisotropy (n = 22 biologically independent cells).[3] In HeLa cells, the mean stagRFP (FRET acceptor) anisotropy decreases in response to Fsk/IBMX stimulation in anisotropy imaging. The mean ratio ± SEM is displayed.

**Supplementary Figure 4**

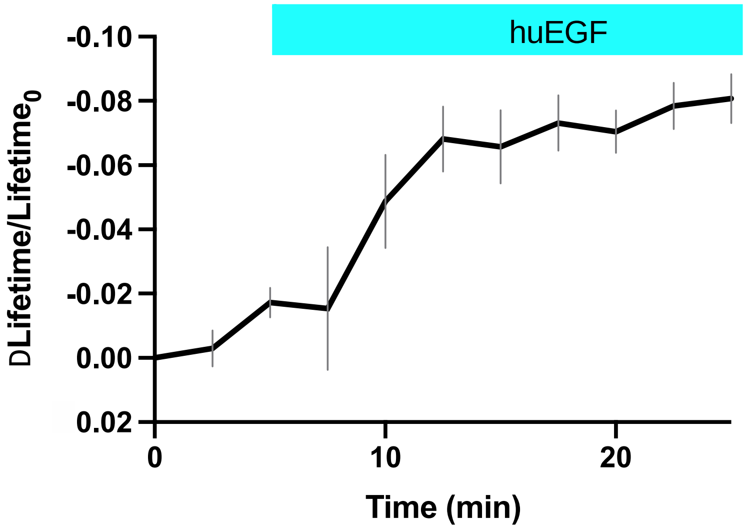


**Supplementary Figure 4.** Response of the fRR-EKARev biosensor is detected as a decrease in the donor lifetime using FLIM-FRET (n = 6 biologically independent cells). In HeLa cells, the mean stagRFP (FRET donor) lifetime decreases in response to huEGF stimulation (100 ng/mL) in Fluorescence Lifetime Imaging Microscopy (FLIM). The mean ratio ± SEM is displayed.

**Supplementary Figure 5**

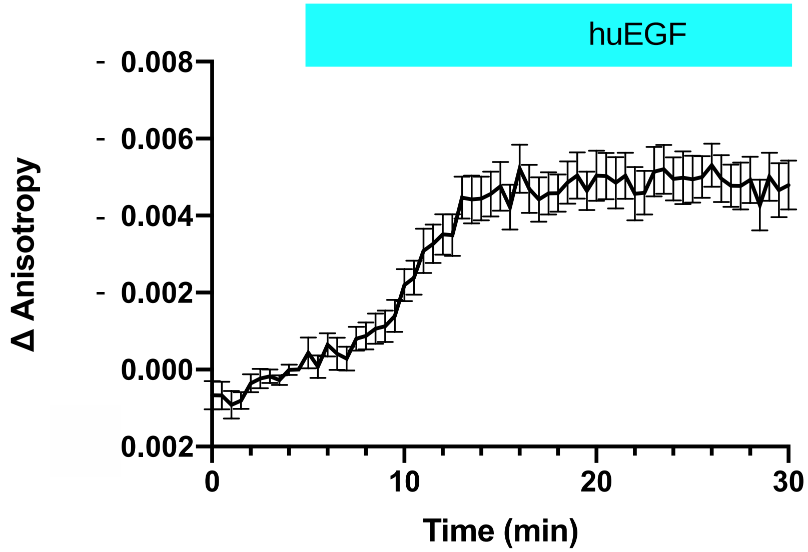


**Supplementary Figure 5.** Response of the fRR-EKARev biosensor is detected as an apparent decrease in the acceptor anisotropy (n = 24 biologically independent cells).[3] In HeLa cells, the mean smURFP (FRET acceptor) anisotropy decreases in response to huEGF stimulation in anisotropy imaging. The mean ratio ± SEM is displayed.

## Supplementary Figure 6

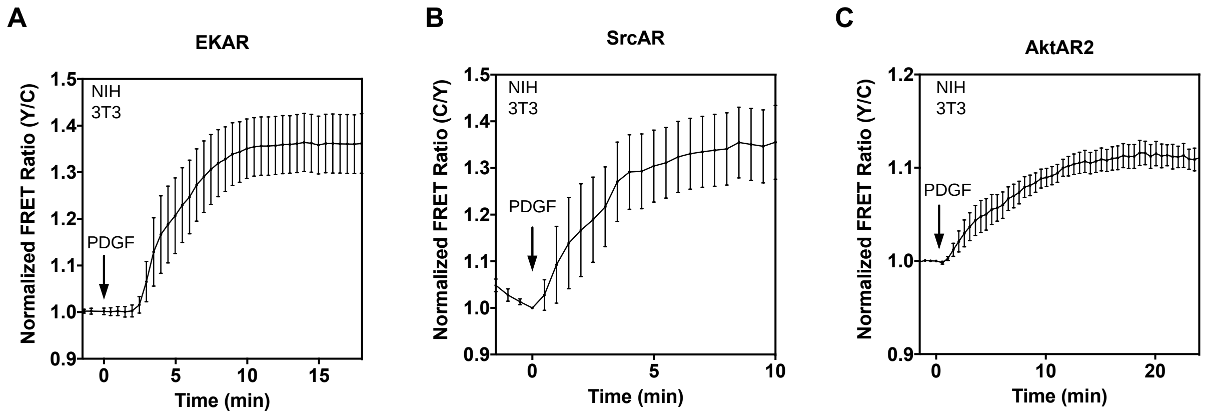


**Supplementary Figure 6.** Supporting control experiments to demonstrate the responses to 50 ng/mL PDGF stimulation observed by multiplex biosensors are consistent with those of known biosensors alone. (A) PDGF stimulated ERK activity in NIH 3T3 cells is captured by the published reporter EKAR (n = 8 biologically independent cells). (B) PDGF stimulated Src activity in NIH 3T3 cells is captured by the published reporter SrcAR (n = 4). (C) PDGF stimulated Akt activity in NIH 3T3 cells is captured by the published reporter AktAR2 (n = 5). The mean ratio ± SEM is displayed in all panels.

**Supplementary Figure 7**

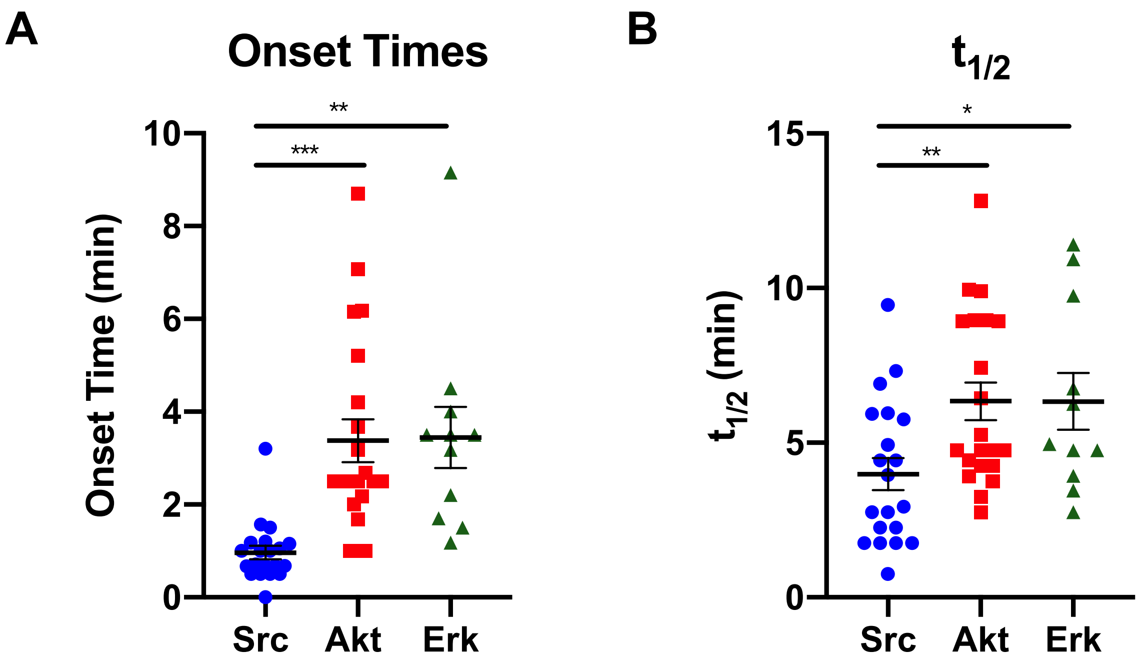


**Supplementary Figure 7.** Measurements of Src, Akt, and Erk kinetics. (A) Comparison of onset times of Src (n = 20 biologically independent cells), Akt (n = 21), and Erk (n = 11) activities in response to 50 ng/mL PDGF stimulation. Results of two tailed, unpaired Welch's t-tests of onset time of Akt against Src ($p< 0.0001$, $t=4.975$, $df=23.88$) and onset time of Erk against Src ($p = 0.0036$, $t=3.688$, $df=10.98$). (B) Comparison of $t_{1/2}$, the time to half maximal value, of Src, Akt, and Erk responses. The mean ± SEM is displayed. Results of two tailed, unpaired Welch's t-tests of $t_{1/2}$ of Akt against Src ($p = 0.005$, $t=2.948$, $df=38.35$) and $t_{1/2}$ of Erk against Src ($p = 0.0402$, $t=2.227$, $df=16.53$). * $p < 0.05$; ** $p < 0.01$; *** $p < 0.001$. Source data are provided as a Source Data file.

**Supplementary Figure 8**

## A. Multiplexed Filter Set A

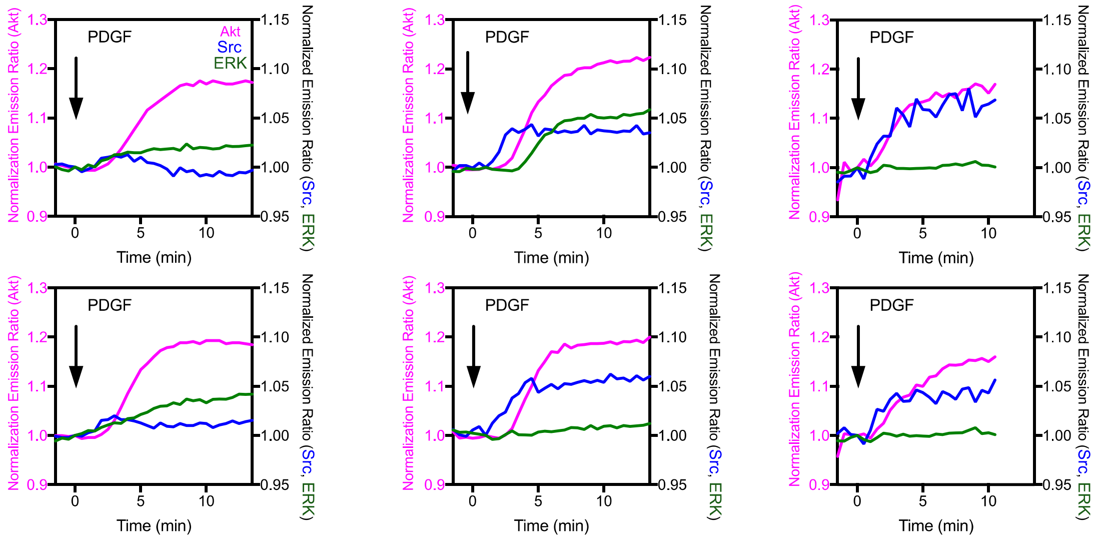


## B. Multiplexed Filter Set B

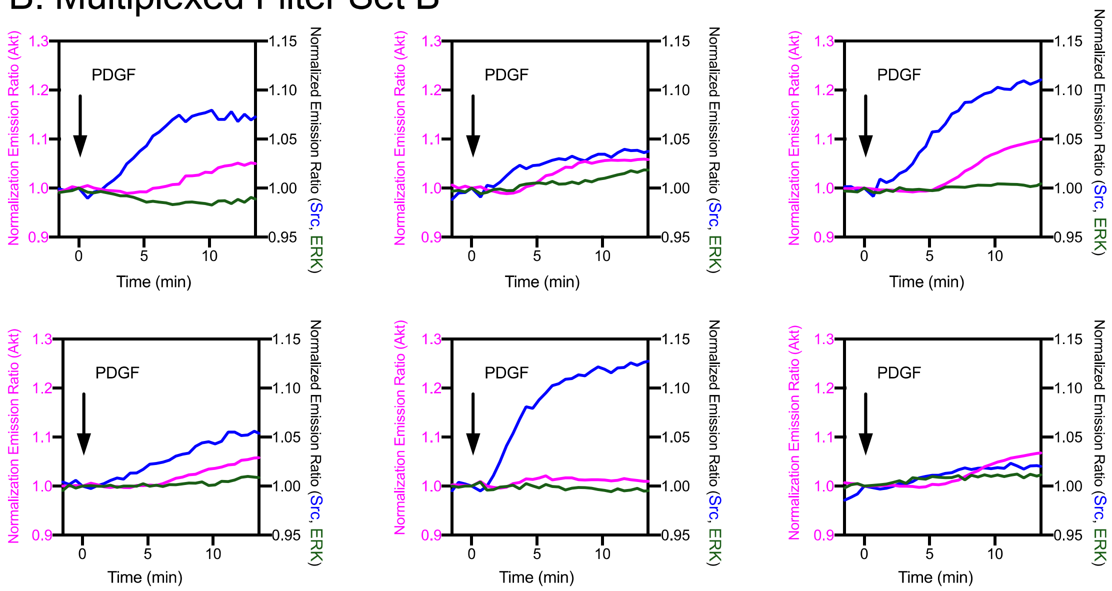


**Supplementary Figure 8.** Representative, individual traces of Src, Akt, and ERK responses to 50 ng/mL PDGF stimulation in single cells using (A) Multiplexed Filter Set A or (B) Multiplexed Filter Set B (see Methods). Although the dynamic range of individual biosensors vary depending on the excitation and emission filters, these biochemical activities are observed in single cells.

# Supplementary Figure 9

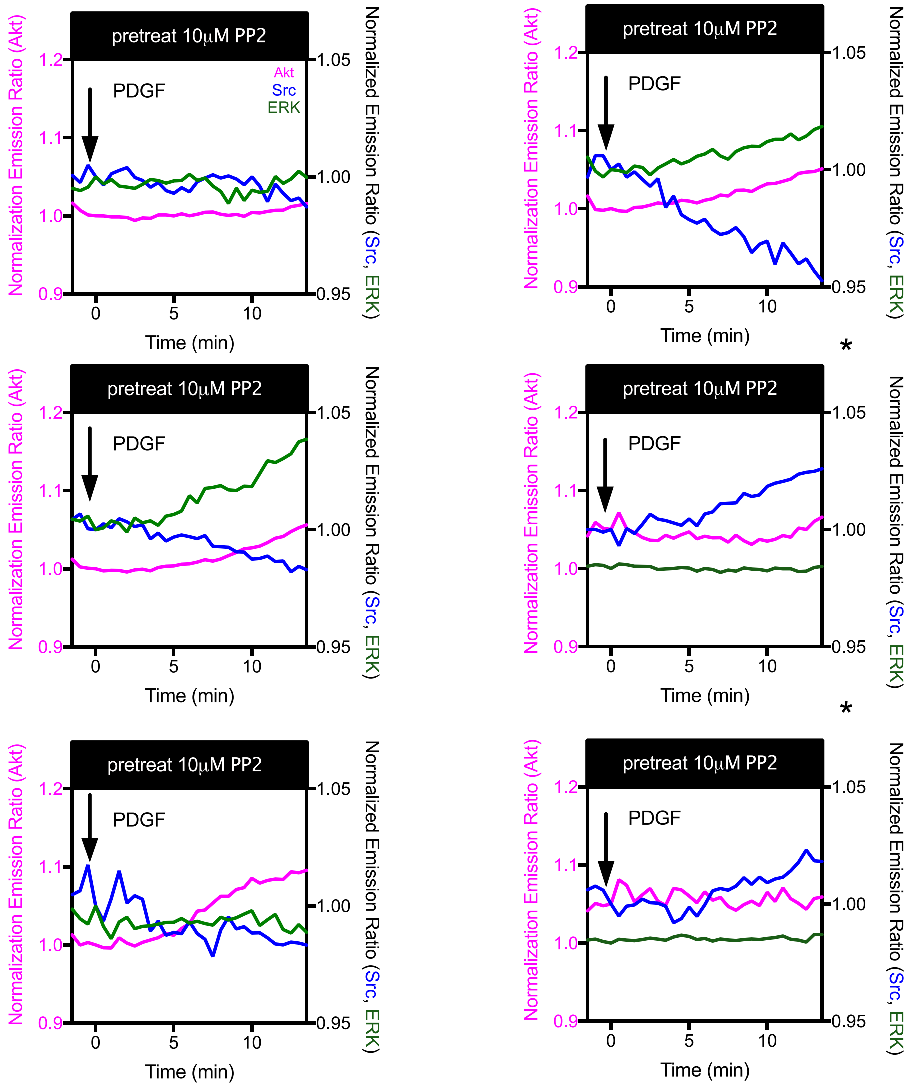


**Supplementary Figure 9.** Individual traces of Src, Akt, and ERK responses to 50 ng/mL PDGF stimulation in single cells pretreated with 10 μM of the Src inhibitor, PP2. Traces marked with * were obtained with Multiplexed Filter Set B and unmarked traces were obtained with Multiplexed Filter Set A.

## Supplementary Figure 10

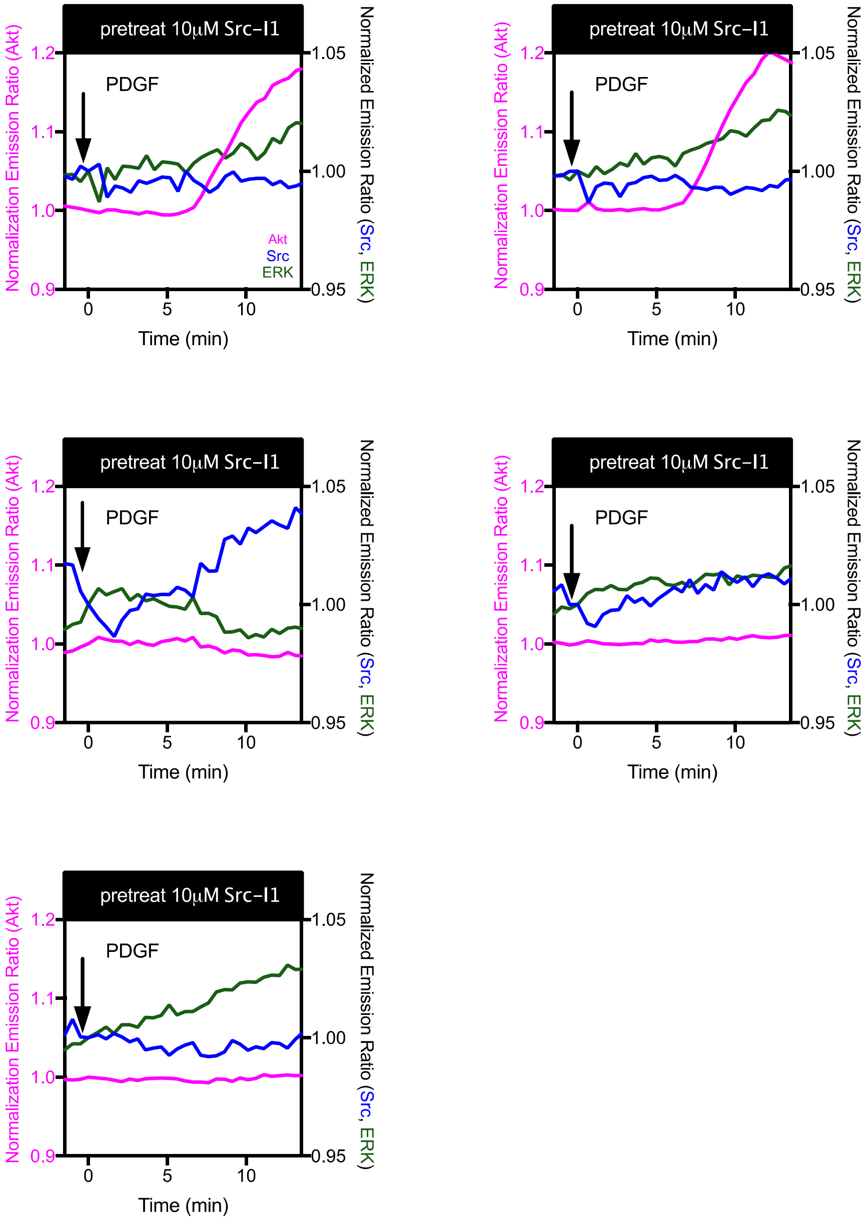


**Supplementary Figure 10.** Individual traces of Src, Akt, and ERK responses to 50 ng/mL PDGF stimulation in single cells pretreated with 10 μM of the Src inhibitor, Src-I1. All traces were obtained with Multiplexed Filter Set B.

**Supplementary Table 1**

| | |
|---|---|
| UTAGT_D159X | GGCCTGGAAGGCAGAACCNNNATGGCCCTGAAGCTCGTG |
| UTAGT_D196X | CCCGGCGTCTACTATGTGNNNCACAGACTGGAAAGAATC |
| UTAGT_R198X | GTCTACTATGTGGACCACNNNCTGGAAAGAATCAAGGAG |
| UTAGT_R157X | GACGGCGGCCTGGAAGGCNNNACCGACATGGCCCTGAAG |
| UTT_D159V | GCCTGGAAGGCAGAACCGTCATGGCCCTGAAGCTCGT |
| TAGRFP_SACI_F | CCCGAGCTCATGAGCGAGCTGATTAAGGAGAACATGCACATGAAGCTG |
| SMURFP_BAMHI_F | CCCGGATCCCATGGCTAAGACTTCCGAACAGAGGGTGAACATTGCTACACT |
| TAGRFP_ECORISTOP_R | GGGGAATTCTCACTTGTGCCCCAGTTTGCTAG |
| SMURFP-DELYK_SPHI-NS_R | GCGATGCATGCGCTTGTACAGCTCGTCGCTCATAGCCTTAATAATGTAATC |
| EKAR_BGL2_F | CCCAGATCTATGGCGGACGAGGAGAAGCTGCC |
| CDC25C_KPNI_R | GGCGGTACCCGGAAATTGGAATGACAGCTTTGCTTTATC |
| CytERM_stagRFP_overhang_fwd | ggcccgggatccaccggtcgccaccatgagcgagctgattaagga |
| CytERM_stagRFP_overhang_rev | gattatgatctagagtcgcggccgcttacttgtgccccagtttgc |

**Supplementary Table 1.** A list of major primers utilized in this work.

**Supplementary References**


1. Costantini, L. M., Fossati, M., Francolini, M. & Snapp, E. L. Assessing the Tendency of Fluorescent Proteins to Oligomerize Under Physiologic Conditions. *Traffic* **13**, 643–649 (2012).
2. Cranfill, P. J. *et al.* Quantitative assessment of fluorescent proteins. *Nat. Methods* **13**, 557–562 (2016).
3. Piston, D. W. & Rizzo, M. A. FRET by Fluorescence Polarization Microscopy. *Methods Cell Biol.* **85**, 415–430 (2008).
