# Supplementary material for: A rationally enhanced red fluorescent protein expands the utility of FRET biosensors
Source: Nat Commun. 2020 Apr 15;11:1848. doi: 10.1038/s41467-020-15687-x (PMC7160135; doi:10.1038/s41467-020-15687-x)
Supplement: Supplementary file 2 — Reporting Summary [file 41467_2020_15687_MOESM2_ESM.pdf]

nature research

Corresponding author(s): Jin Zhang

Last updated by author(s): Mar 10, 2020

Reporting Summary

Nature Research wishes to improve the reproducibility of the work that we publish. This form provides structure for consistency and transparency in reporting. For further information on Nature Research policies, see Authors & Referees and the Editorial Policy Checklist.

Statistics

For all statistical analyses, confirm that the following items are present in the figure legend, table legend, main text, or Methods section.

n/a	Confirmed	
☐	☒	The exact sample size (*n*) for each experimental group/condition, given as a discrete number and unit of measurement
☐	☒	A statement on whether measurements were taken from distinct samples or whether the same sample was measured repeatedly
☐	☒	The statistical test(s) used AND whether they are one- or two-sided<br>*Only common tests should be described solely by name; describe more complex techniques in the Methods section.*
☒	☐	A description of all covariates tested
☐	☒	A description of any assumptions or corrections, such as tests of normality and adjustment for multiple comparisons
☐	☒	A full description of the statistical parameters including central tendency (e.g. means) or other basic estimates (e.g. regression coefficient) AND variation (e.g. standard deviation) or associated estimates of uncertainty (e.g. confidence intervals)
☐	☒	For null hypothesis testing, the test statistic (e.g. *F*, *t*, *r*) with confidence intervals, effect sizes, degrees of freedom and *P* value noted<br>*Give P values as exact values whenever suitable.*
☒	☐	For Bayesian analysis, information on the choice of priors and Markov chain Monte Carlo settings
☒	☐	For hierarchical and complex designs, identification of the appropriate level for tests and full reporting of outcomes
☒	☐	Estimates of effect sizes (e.g. Cohen's *d*, Pearson's *r*), indicating how they were calculated

Our web collection on statistics for biologists contains articles on many of the points above.

Software and code

Policy information about availability of computer code

Data collection	Raw imaging data were acquired using either METAFLUOR 7.7, Micro-Manager2.0, NIS-Elements, or LAS X (Lecia) software.
Data analysis	Raw imaging data were analyzed using METAFLUOR 7.7, ImageJ, LAS X (Lecia) software, or custom MATLAB scripts that are available upon request. Statistical analyses were performed using Excel or GraphPad Prism 7 and 8.

For manuscripts utilizing custom algorithms or software that are central to the research but not yet described in published literature, software must be made available to editors/reviewers. We strongly encourage code deposition in a community repository (e.g. GitHub). See the Nature Research guidelines for submitting code & software for further information.

Data

Policy information about availability of data

All manuscripts must include a data availability statement. This statement should provide the following information, where applicable:
- Accession codes, unique identifiers, or web links for publicly available datasets
- A list of figures that have associated raw data
- A description of any restrictions on data availability

Data in figures will be available from the corresponding author after publication upon reasonable request.

Field-specific reporting

Please select the one below that is the best fit for your research. If you are not sure, read the appropriate sections before making your selection.

☒ Life sciences ☐ Behavioural & social sciences ☐ Ecological, evolutionary & environmental sciences

For a reference copy of the document with all sections, see nature.com/documents/nr-reporting-summary-flat.pdf

Life sciences study design

All studies must disclose on these points even when the disclosure is negative.

Sample size	No sample-size calculations were performed. Sample sizes were similar to those generally employed in the field.
Data exclusions	Cells that did not express all of a given set of constructs (for multi-parameter imaging experiments) were excluded from analysis. All exclusion criteria were pre-determined.
Replication	All experiments were repeated at least three times; all replication attempts were successful.
Randomization	No animal or human studies were involved; no randomization has been performed.
Blinding	No animal or human studies were involved; no blinding was used for data analysis.

Reporting for specific materials, systems and methods

We require information from authors about some types of materials, experimental systems and methods used in many studies. Here, indicate whether each material, system or method listed is relevant to your study. If you are not sure if a list item applies to your research, read the appropriate section before selecting a response.

Materials & experimental systems

n/a	Involved in the study	
☒	☐	Antibodies
☐	☒	Eukaryotic cell lines
☒	☐	Palaeontology
☒	☐	Animals and other organisms
☒	☐	Human research participants
☒	☐	Clinical data

Methods

n/a	Involved in the study	
☒	☐	ChIP-seq
☒	☐	Flow cytometry
☒	☐	MRI-based neuroimaging

Eukaryotic cell lines

Policy information about cell lines

Cell line source(s)	All cell lines were obtained from ATCC.
Authentication	Cell line identities were verified via STR analysis by the commercial source and then maintained separately and isolated from one another to avoid cross-contamination.
Mycoplasma contamination	All cell lines were determined to be free of mycoplasma contamination based on regular DNA staining.
Commonly misidentified lines (See ICLAC register)	HEK cells were used in some experiments. However, the identity of the cells was not central to the outcome of these experiments.
